# State-of-the-art Review on the Antiparasitic Activity of Benzimidazole-based Derivatives: Facing Malaria, Leishmaniasis, and Trypanosomiasis

**DOI:** 10.2174/0929867331666230915093928

**Published:** 2024-02-26

**Authors:** Valeria Francesconi, Marco Rizzo, Silvia Schenone, Anna Carbone, Michele Tonelli

**Affiliations:** 1 Department of Pharmacy, University of Genoa, Viale Benedetto XV, 3, Genoa, 16132, Italy

**Keywords:** Protozoan infections, antiprotozoal agents, benzimidazole-based derivatives, antimalarial compounds, antileishmanial compounds, antitrypanosomal compounds

## Abstract

Protozoan parasites represent a significant risk for public health worldwide, afflicting particularly people in more vulnerable categories and cause large morbidity and heavy economic impact. Traditional drugs are limited by their toxicity, low efficacy, route of administration, and cost, reflecting their low priority in global health management. Moreover, the drug resistance phenomenon threatens the positive therapy outcome. This scenario claims the need of addressing more adequate therapies. Among the diverse strategies implemented, the medicinal chemistry efforts have also focused their attention on the benzimidazole nucleus as a promising pharmacophore for the generation of new drug candidates. Hence, the present review provides a global insight into recent progress in benzimidazole-based derivatives drug discovery against important protozoan diseases, such as malaria, leishmaniasis and trypanosomiasis. The more relevant chemical features and structure-activity relationship studies of these molecules are discussed for the purpose of paving the way towards the development of more viable drugs for the treatment of these parasitic infections.

## 1 INTRODUCTION

Parasitic diseases are still a plague that has a huge impact on public health and the overall social and economic well-being of affected countries, mainly developing countries. The principle human diseases caused by protozoa, including malaria, leishmaniasis and trypanosomiasis, are responsible for 790 million cases of morbidity, with an estimated 810,000 death cases annually [1]. Most parasitosis are neglected, like leishmaniasis and trypanosomiasis, since they afflict the world’s poorest populations and still do not receive the proper attention from the funding agencies, thus, research and health programs in this field remain underdeveloped [2].

The global burden of parasitic diseases is generally worsened by the absence of marketed vaccines. RTS, S/AS01 (Mosquirix™) is the first licensed malaria vaccine for *P. falciparum* to be widely recommended by WHO for children at risk; a two years immunization pilot program in Ghana, Kenya, and Malawi [3] has given positive results in terms of safety and efficacy, lowering the disease severity and the hospitalization rate of pediatric population with malaria infection. However, some concerns have arisen due to the short life time of immunity induced by the vaccine as well as the high production costs [4, 5]. Another vaccine candidate (R21/Matrix-M) has entered a phase 3 clinical trial (ClinicalTrials.gov Identifier: NCT04704830), and if it is approved, it would be an additional resource for the global supply. Despite this, the development of new safe and effective agents may also help manage protozoan diseases, especially for those infections where the drugs are the only weapons for prophylactic and therapeutic purposes. Most of the antiparasitic drugs are old drugs whose use for a long time has induced the emergence of parasite drug resistance [6]; the threats are indeed increasing, especially since epidemiological surveillance is coming from several countries, making the scenario more serious and complex. Hence, the identification of new drugs to treat Malaria, Leishmaniasis, and Trypanosomiasis is always challenging.

### 1.1 Malaria

Malaria is a life-threatening disease caused by *Plasmodium* parasites that are transmitted to people through the bites of infected female *Anopheles* mosquitoes. Five parasite species cause malaria in humans, and in particular, *P. falciparum* and *P. vivax* are the most worrisome for public health. *P. falciparum* is the deadliest parasite and the most prevalent throughout the African continent, while *P. vivax* is the most widespread outside of sub-Saharan Africa. Recent estimates suggest that 247 million clinical cases of malaria occur every year globally, with 619,000 deaths reported in 2021 [7].

The observed increase in malaria cases with respect to pre-pandemic years could be explained by the significant COVID-19 disease impact that has shifted most of the public health initiatives to contain the emergency crisis. To make matters worse, there have been long-running humanitarian crises and the repercussions of climate change on the spread of the parasitic disease [7].

The life cycle of malarial parasites starts with the growth of *Plasmodium* in an insect host intermediate that, during a blood meal, deposits it as sporozoite form into the bloodstream of a final host, like a human being. In the host’s liver, sporozoites evolve into merozoites by asexual development, entering the systemic circulation and invading red blood cells, causing the clinical manifestation of the disease. Characteristically, in *P. vivax* and *ovale* infections, some parasites may remain latent in the liver (hypnozoite reservoir) and then reactivate to produce blood-stage infections [8]. In erythrocytes, most parasites asexually reproduce from ring forms to trophozoites and lastly to mature schizonts. According to the different Plasmodium species, the schizonts-induced lysis of erythrocytes releases a variable number of merozoites. A group of asexual merozoites can then differentiate into male and female gametocytes that constitute the transmissible form of the parasite to an insect vector. After an infected blood-feeding, the gametocytes reach the mosquito’s midgut and form the zygotes that evolve into oocysts; they successively develop into a new progeny of sporozoites, thus restarting the infective cycle [4].

The arsenal of antimalarial drugs can be divided into three main classes, such as quinoline derivatives (chloroquine and analogues), antifolates (pyrimethamine, proguanil plus atovaquone), and artemisinin derivatives (artemether) and some distinct antibiotics [9], that are used in monotherapy or in combination chemotherapy.

*Plasmodium* pathogens have developed resistance to conventional therapeutics like chloroquine (CQ) and antifolates. Drug resistance phenomenon even to the artemisinin derivatives and artemisinin-based combination therapy (ACT) has been observed in several areas of Africa [7] and southeast Asia, arousing a great concern [10, 11].

The emergence of resistance has stimulated the modification of existing antimalarial scaffolds and the search for a new generation of medicines characterized by innovative mechanisms of action. Thus, many promising compounds have been tested in monotherapy or, more effectively, in combination therapies (particularly ACT) with the scope to improve efficacy and delay the onset of resistance. Different strategies are being pursued, as illustrated and discussed in some recent literature examples [12-15].

For most of the marketed antimalarials, the mechanism of action is still to be unravelled as they exhibit multitargeting behaviour. This concept is relevant in the context of multifactorial diseases, such as malaria and other vector-borne parasitosis, that are based on intricate interactions among pathogens, vectors, reservoir hosts, and environmental factors [16]. The different initiatives have not yet solved the problem of breaking the chain between the parasite reservoir in animals and infections in man. Therefore, the huge economic losses and serious damage to social well-being in endemic countries continue to be a heavy burden.

### 1.2 Neglected Tropical Diseases (NTDs)

NTDs represent a group of 20 different conditions widespread in 150 countries and affect about one billion people. They are caused by parasites, viruses, bacteria, fungi, and toxins [17]. Among protozoan parasites, trypanosomatids are the etiologic agents of NTDs, such as leishmaniasis, Human African Trypanosomiasis (HAT), and Chagas disease, causing significant morbidity and mortality in humans and animals.

#### 1.2.1 Leishmaniasis

Leishmaniasis is particularly common, with an estimated 700000 to 1 million infections occurring each year [18]. It is caused by kinetoplast protozoans of the *Leishmania* genus, which are transmitted to mammalian hosts by the bite of the infected female phlebotomine sandflies. Over 20 *Leishmania* and over 90 sandfly species are known as of today. The parasites enter host cells as flagellated and motile promastigote forms which are phagocytosed by macrophages. The promastigotes differentiate into non-motile amastigote forms inside the phagosome of the host cells. Subsequent fusion with lysosomes leads to phagolysosomes in which *L.* parasites can proliferate and survive in the adverse environment. Macrophages are also the major effector cells able to eradicate the infection by activating a suitable immune response. Three main clinical manifestations of *L*. disease, depending on infecting species and human immune response, are reported: cutaneous (CL), mucocutaneous (MCL), and visceral kala-azar (VL) [19]. CL is the most frequent form and produces mainly skin ulcers that often mend spontaneously, although in some cases cause serious disability or stigma; MCL is the most deforming type affording to partial or total damage of nose, mouth and throat mucous membranes; VL represents the most dangerous form leading to death in over 95% of cases; if untreated, it results in variable fever, weight loss, enlarged liver and spleen, and anaemia [20]. The available drugs for the *L.* diseases include pentavalent antimonials (sodium stibogluconate and meglumine antimoniate) as conventional therapy and liposomal amphotericin B, pentamidine salts, paromomycin sulfate, and miltefosine as second-line drugs. These agents are characterized by different chemical scaffolds, which lead to several mechanisms of action. Unfortunately, the current treatments show severe side effects, drug resistance development, poor potency, and high cost. Vaccines are unavailable for human *L.* infections so far and only two vaccines are licensed in Europe for canine leishmaniosis (CanL) (CaniLeish^®^ and LetiFend^®^) [21]. CanL is a sand fly-borne incurable pathology caused by *L. infantum*. It is a major zoonosis endemic in >89 countries, with an increase of imported cases in non-endemic zones, resulting in a serious public health issue. Unfortunately, remission is hardly achieved. The recurrences are common, and above all, the infected dogs represent the major reservoir of the protozoan for humans [22]. Although 50% of the infected dogs are asymptomatic, CanL is a chronic and multisystemic disease able to affect all organs. The above-mentioned vaccines offer a low protective effect. Beyond the use of topical insecticides, the first-line treatment is the combined use of human anti-leishmanial agents (pentavalent antimonials or miltefosine) with allopurinol. The development of new effective anti-leishmanial compounds is complex because the efficacy varies depending on parasite species and clinical manifestations. Besides, they have to pass three membranes to kill the intracellular amastigotes, leaving the macrophage hosts unaffected. In the last years, drug discovery in the leishmaniasis field has significantly improved due to the efforts of international consortia funded by governments and charities. In this scenario, the Drugs for Neglected Diseases *initiative* (DNDi), with its partners, published a target product profile (TPP) in which the minimal and optimal requisites for a new anti-leishmanial agent were detailed [23, 24]. In particular, a new agent for VL infections should be orally administered, show a good safety profile during pregnancy and in immune-deficient patients, reduce parasite burden by 95% in a short treatment, and be inexpensive.

#### 1.2.2 Trypanosomiasis

*Trypanosoma brucei* is the causative agent of HAT or “sleeping sickness,” and *Trypanosoma cruzi* causes Chagas Disease. In particular, HAT is caused by two subspecies of *T. brucei*, which are morphologically indistinguishable but produce different disease courses in infected hosts: *T. b. rhodesiense* is endemic in eastern and southern Africa and leads to an acute disease progression, whereas *T. b. gambiense* is associated with a chronic form of the disease and is prevalent in Western and central Africa. To date, control efforts have drastically decreased the spread of this infection; however, sleeping sickness still represents a public health. WHO has set the target to achieve the complete eradication of HAT before 2030, seeking cooperation among multiple sectors based on the *One Health* approach. Indeed, diagnosis and tracking of the cases, together with the requirement of drugs and facilities to treat affected people, are strictly linked with the need for an efficient control system of vector spreading and of animal reservoirs [25]. Nowadays, there are five drugs approved for the treatment of HAT: suramin and pentamidine, administered in the first stages of the infection (hemo-lymphatic); eflornithine and melarsoprol, recommended for the second stage (meningo-encephalic). Recently, the first-line treatment options have been expanded to fexinidazole, which has been approved by the European Medicines Agency (EMA) for the treatment of *T. b. gambiense* infections, as a daily dose (for 10 days) against both disease’s stages [26]. However, these drugs are generally toxic and difficult to administer, and they are also prone to develop drug resistance. Thus, the research on new molecules is still intense. *T. cruzi* is the causative agent of Chagas disease, which is endemic in Latin America. WHO estimates that about 6-7 million people worldwide are infected with *T. cruzi*. Disease control is a challenging task as conventional therapy, based on benznidazole or nifurtimox, is effective only in the early phases of post-infection. The access to early diagnosis is often delayed since the acute symptoms immediately following the infection are either absent or mild and non-specific. Moreover, in endemic countries an efficient system capable of detecting and tracking the disease at onset is lacking; therefore, Chagas disease is often addressed as a “silent and silenced disease” still representing a public health concern [27].

Both *T. brucei* and *T. cruzi* are heteroxenous parasites, exploiting an insect vector to finalise their life and reproductive cycle [28]. Tsetse flies (*Glossina* spp) represent the intermediate hosts of *T. brucei*, while *T. cruzi* is mainly transmitted by contact with faeces/urine of infected bugs of the triatomine family. Trypanosomatids are hence transferred to the final vertebrate hosts during the insect´s blood meal [29]. Alongside the heteroxenous life cycle entailing a definitive host and an intermediate host (indirect life cycle), parasites belonging to the *Trypanosomatidae* family also share some peculiar structural characteristics, namely a single flagellum and a kinetoplast, an organelle containing a large massed mitochondrial DNA [28].

Trypanosomatids can exist in different forms depending on the stage of their life cycle; *T. brucei* differentiates into two bloodstream forms in the mammalian host: a replicative long slender trypomastigote (LSF) or a short stumpy trypomastigote form (SSF). The LSF could evade the immune system surviving inside the host, while the SSF is the tsetse-infective form. In the vector midgut, the parasites assume the epimastigote proliferative forms before evolving to the non-proliferative metacyclic trypomastigotes, which are infective for mammalian cells. Metacyclic trypomastigotes differentiate into amastigotes within the host cells and replicate; then, the intracellular amastigotes assume the form of non-replicative trypomastigotes that cause host cell lysis. Non-replicative bloodstream trypomastigotes could re-enter the triatomine vector midgut and regenerate epimastigotes [29].

## 2 PROMISING DRUG TARGETS

**Fig. (1):**
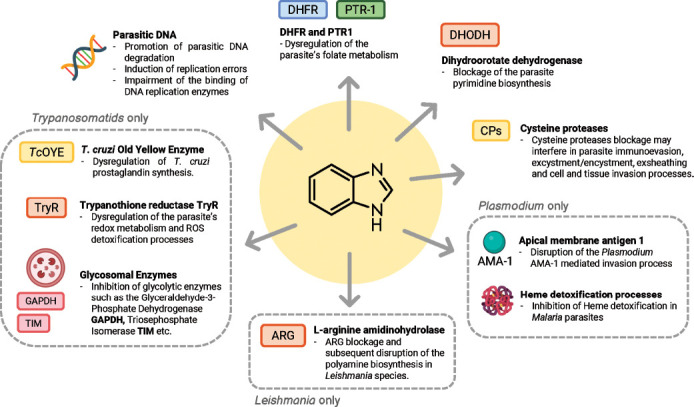
Promising drug targets in malaria, leishmaniasis and trypanosomiasis.

The following chapters focus on the most investigated protozoan targets and the state of the art of the related benzimidazole-based inhibitors thus far identified (Fig. **1**). Noteworthy, this class of antiparasitic agents displays a wide range of biological actions, sometimes resembling those of approved antiprotozoan drugs or providing new therapeutic properties with the prospect that even more innovative drugs could be introduced in the years to follow.

Several conventional drugs bear a benzimidazole ring as their main moiety or key substructure [30, 31]. Isostere of purine bases, the benzimidazole nucleus continues to draw interest in drug discovery, owing to its important biological significance in different pharmacological settings [32-34]. Concerning the antimicrobial purposes, such as antibacterial, antiviral and antiprotozoal activities, the literature reports many examples of benzimidazole derivatives [35-37].

Interestingly, the benzimidazole derivatives are often screened against diverse parasites, with a view to disclosing broad-spectrum antiprotozoal agents underlying possible common mechanisms of parasite growth inhibition. However, the study of the benzimidazole chemical space has, till now, led to the identification of hit/lead compounds, reaching only a few of them to the preclinical profiling stage in animal models of parasitic infections.

### 2.1 Malaria Targets

Several promising targets are emerging in the fight against malaria, such as dihydroorotate dehydrogenase (DHODH) and apical membrane antigen 1 (AMA-1). Moreover, agents binding to the minor groove of DNA and eme detoxification process inhibitors showed encouraging results (Fig. **1**).

#### 2.1.1 DNA Minor Grove

Like pentamidine, which is a bisamidinium compound at physiological pH, dicationic bisbenzamidines and structurally related heterocycles have long been known as antiparasitic DNA-targeting non-covalent agents [38, 39]. They prevalently accumulate in the mitochondrial kinetoplast of trypanosomatids rather than in the nucleus, as it occurs for *Plasmodium* genus [40, 41]. They bind to minor DNA grooves and cause replication errors by impairing the activity of DNA-dependent enzymes and DNA degradation, hence cell death. The protozoan DNA architecture is unusually rich in thousands of AT-repeated sequences, which are specifically recognized by diamidines when they adopt an appropriate curved shape and exhibit HBD NH groups taking contacts with N(3) of adenine and O(2) of thymine base pairs. As human DNA does not demonstrate such an arrangement, the analysis of the interspecies activity profile of this class of molecules revealed a decrease in the DNA binding capacity passing from protozoa to human cells. Therefore, they were expected to exert no toxic effects at the therapeutic concentration but only at higher doses [38, 41].

#### 2.1.2 Dihydroorotate Dehydrogenase (DHODH)

An important enzyme implicated in DNA biosynthesis is DHODH (Fig. **1**), promoting the Flavin mononucleotide-dependent formation of orotic acid, an intermediate product in the biosynthesis of pyrimidine bases for parasite survival [9, 42]. Although *Plasmodium falciparum* (*Pf*) DHODH belongs to class 2 DHODH together with human isoform, significant differences between the two enzymes were elucidated [42, 43], making the design of selective DHODH inhibitors possible. *Pf* DHODH inhibitors preferentially bind to a hydrophobic pocket, lined by the two N-terminal α-helices 1 and 2 and the α/β barrel domain, whose shape and flexible structure frame the main points of divergence to human ortholog, while the hydrogen-bond site (Arg265 and His185) is a conserved region across all class 2 DHODH isoforms [42]. Contrarily to parasites, humans may count on the salvage pathway as a bypass for pyrimidine biosynthesis. Thus, DHODH targeting agents may exert a limited impact on essential human functions. Therefore, this enzyme represents a validated target for addressing anti-infective agents to be applied in different pharmacological settings [42].

#### 2.1.3 Apical Membrane Antigen 1 (AMA-1)

During the asexual blood stage, sporozoites and merozoites of *Plasmodium* species share a complex array of proteins to invade hepatocyte and erythrocyte host cells [44, 45], respectively, for their intracellular replication cycle. AMA-1 is an antigen on the surface of the invasive parasites, which represents the main target of naturally acquired antibodies in malaria-endemic populations, thus suggesting this protein as an important tool to set-up a vaccine [46]. Furthermore, AMA-1 is recognised as a validated target for the development of small molecules [45], enabling them to block the invasion and the growth of *P*. parasites' asexual blood stages (Fig. **1**). Indeed, only recent studies are somehow solving the complexity of the AMA-1-mediated invasion process, pointing out that diverse pathways may occur within the *Plasmodium* genus [47], thus burdening the development of broad-spectrum antimalarial therapeutics.

#### 2.1.4 Heme Detoxification

*Plasmodium* species spend much of their life as intracellular parasites, thus remaining safe in the host cell environment and evading the host's immune response. During infection of the human host, malaria parasites invade red blood cells and, in the digestive vacuole, metabolize a large amount of haemoglobin releasing free heme that can be toxic. Then, heme undergoes oxidation of Fe^2+^ to Fe^3+^ by molecular oxygen forming hematin (protoporphyrin IX), which finally dimerizes into non-toxic hemozoin crystals [48, 49]. The inhibition of this detoxification process (Fig. **1**), based on heme catabolism, increases its accumulation in the parasite, leading to toxicity and death of the parasite. This mechanism of action is characteristic of 4-aminoquinoline-based antimalarial drugs [13], such as chloroquine, lumefantrine, quinine and mefloquine, which are usual reference compounds for biological testing.

#### 2.1.5 Other Druggable Targets

Another critical step of the malaria parasites' life cycle is the egress event, allowing them to leave the host and promote their dissemination. A lot of parasite enzymes take part in this process, including proteases, kinases, phospholipases, and other proteins. Interestingly, all these proteins are eligible targets for the design of innovative antimalarial medicines [50-52].

### 2.2 Trypanosomiasis and Leishmaniasis Targets

Trypanosomatids, besides sharing the typical eukaryotic organelles, also possess some unique biological structures and hallmark enzymes that could represent promising targets for drug design.

#### 2.2.1 ROS/RNS Detoxification

The kinetoplast is a highly specialized region of the parasite mitochondrion which contains a peculiar DNA. The kinetoplast DNA (kDNA) is organized in an intricate architecture of thousands of circular DNA molecules forming a single supramolecular structure that encodes for rRNAs and proteins of the respiratory chain or guide RNAs involved in the RNA editing processes [53]. kDNA could be subject to the attack of reactive oxygen and nitric species (ROS/RNS) produced during mitochondrial respiration by complexes I-IV. At low concentrations, these reactive species are crucial to the parasite cell physiology, involving cellular signalling or cytotoxicity. On the contrary, high levels of ROS/RNS can threaten parasite survivability [54], interfering with several biosynthetic pathways and damaging proteins and DNA. ROS release is also described as one of the defence mechanisms employed by the mammalian host immune system (macrophages) to contrast the infection. As a part of their response to reduce ROS, trypanosomatids have developed several detoxification countermeasures arranging a poll of enzymes, such as Fe^-^ superoxide dismutases (SODs), cytochrome c peroxidase (CCP) (in *Leishmania* spp.), and thiol peroxidases, such as 2-Cys-peroxiredoxines, such as the tryparedoxin peroxidase (TXNPx), and the nonselenium glutathione peroxidase (nsGPXA3) [55]. TXNPx is the final enzyme of a redox cascade in which the electrons flow from NADPH to trypanothione [T(S)_2_/T(SH)_2_], a parasitic dimer derivative of glutathione in mammals. Trypanothione is the natural substrate of trypanothione reductase (TryR) (Fig. **1**), a flavoprotein localised in mitochondria, glycosomes and cytosol of parasites [56]. TryR is absent in the mammalian hosts, where it is replaced by glutathione reductase (GR), showing a similar antioxidant functioning. With respect to GR, the TryR binding pocket is large, hydrophobic and exhibits a negative charge, making possible the design of selective inhibitors [57]. However, TryR undergoes a high-rate turnover. Thus, TryR activity should be inhibited by 90% to provide a significant effect on parasite viability [58].

The enzyme converts trypanothione disulfide T(S)_2_ into its reduced form T(SH)_2_, and the electron passes to another protein tryparedoxin (TXN) and finally to the tryparedoxin peroxidase (TXNPx), which catalyses hydroperoxides reduction [59]. These detoxification mechanisms sustain trypanosomatids' survival during the infection since *T. cruzi* TXNPx has been found to be upregulated during the infective host colonization phase. Even if the trypanosomatids thiol peroxidases redox system appears to be a potential target for the development of selective antiparasitic compounds some challenging points need to be stressed: studies on *T. brucei, T. cruzi,* and *L. brasiliensis* enlightened that the inhibition of nsGPXA3 alone is not able to produce an adequate antiparasitic effect, because of the metabolic bypass provided by TXNPx thiol peroxidases; even though they have different preferences for their substrate hydroperoxides, they could compensate each other’s work, allowing parasite survival. Conceivably, dual inhibitors could offer the attained antiparasitic effect [55].

#### 2.2.2 Glycosomal Enzymes

The kinetoplastids are also characterised by a peculiar type of peroxisome-related organelles called glycosomes due to the presence of enzymes in the glycolytic and gluconeogenic pathways. The proteome of these organelles differs among trypanosomatids and depends on the phase of the parasite cycle; it may include glycolytic and gluconeogenesis enzymes (Fig. **1**), such as hexokinase (HXK), glucokinase (GlcK), phosphoglucose isomerase (PGI), phosphofructokinase (PFK), aldolase (ALD), triosephosphate isomerase (TIM), glyceraldehyde-3-phosphate dehydrogenase (GAPDH), phosphoglycerate kinase (PGK) *etc.*

TIM is a glycolytic enzyme (Fig. **1**) contained in trypanosomatid glycosomes that catalyses the reversible interconversion of glyceraldehyde-3-phosphate and dihydroxyacetone phosphate, a key step of the glycolytic pathway. TIM is a homodimer that structurally adopts an α/β barrel folding; each monomer is composed of eight parallel β strands in the centre, surrounded by eight α-helices connected to each other by loops.

Glycosomes also contain enzymes involved in other metabolic pathways: ether-lipid synthesis, β-oxidation, sterol and isoprenoid synthesis, purine salvage, pyrimidine synthesis, amino acid metabolism and defence against oxidative stress [60].

#### 2.2.3 Old Yellow Enzyme (OYE)

OYE (Fig. **1**) is an oxidoreductase involved in the metabolic pathway of arachidonic acid (AA) of *T*. *cruzi* and *L.* parasites [61]. OYE catalyses prostaglandin F2α (PGF2α) synthesis and is also involved in the reduction of trypanocidal drugs, such as nifurtimox. PGF2α synthases play a key role in the trypanosomatids infective cycle. Thus, they are present in all the members of the trypanosomatidae family. Commonly, the parasite PGF2α synthases belong to the Aldo-Keto reductase protein family. *T. cruzi* diverges from this pattern since its aldo-keto reductase (TcAKT) is not capable of catalysing the PGH_2_ reduction to PGF2α; thus, it encodes a member of the OYEs family to carry out this reaction. OYE is a cytosolic flavoprotein NADPH oxidoreductase that can reduce nitro esters, nitroaromatics, and α,β-unsaturated compounds and is also involved in hydrogen peroxide detoxification [62].

#### 2.2.4 L-arginine Amidinohydrolase (ARG)

In *Leishmania* parasites, ARG (Fig. **1**) is involved in the polyamine biosynthesis by supplying L-arginine from the host. After L-arginine hydrolysis by ARG, its metabolic product, L-ornithine, is released into the cytosol and converted into putrescine by the action of ornithine decarboxylase (ODC). Finally, spermidine is obtained through the transfer of an aminopropyl group from decarboxylated S-adenosylmethionine (dsSAM) to a terminal amino group of putrescine by spermidine synthase (SPDSYN). The polyamines play an important role in the parasite survival and proliferation in the host cells. Moreover, the spermidine binds two glutathione residues, leading to trypanothione, which is implicated in the redox homeostasis of the parasite.

Interestingly, the enzyme shares 39-43% identity with human ARG [63, 64]. Instead, a 75.8% identity was observed by the alignment of ARG sequences from different *L*. species, with no variations concerning the active site and the metal binding regions. The variability of the compound binding affinity was attributed to amino acid differences outside the active site pocket.

In response to Th1 cytokines, the macrophages induce nitric oxide synthase (NOS) activity to produce L-citrulline and cytotoxic nitric oxide (NO) starting from L-arginine. Thus, the deprivation of this AA from the host can promote the parasite's survival and proliferation. Dissimilarity in L-arginine metabolism in cutaneous and visceral promastigote species was disclosed, reporting that *L. mexicana* and *L. major* utilize more L-arginine than *L. donovani,* biosynthesising a larger amount of L-ornithine and L-arginine metabolites [65]. Moreover, the *L. donovani* ARG was essential for ornithine and polyamine synthesis, but ODC represented the rate-limiting enzyme for polyamine production [66].

#### 2.2.5 Cysteine Proteases (CPs)

CPs (Fig. **1**) play a pivotal role in protozoa virulence, proliferation, and metabolism [67]. They are highly expressed in *L.* species and are structurally different from the mammalian homologous cathepsins. *L. mexicana* possesses three types of CPs belonging to the papain family: CPA and CPB (cathepsin L-like) and CPC (cathepsin B-like) [68]. In particular, the isoform CPB2.8 was extensively investigated as an effective antileishmanial target, and few reports described inhibitors of *L. mexicana* CPB2.8∆CTE, which represents the recombinant form of the CP2.8 isoform expressed without the C-terminal extension. *L. mexicana* CPB-deficient mutants were found to be less able to infect macrophages and induce lesions in BALB/c mice than wild-type parasites. In general, the virulence of *L. mexicana* was associated with the CPB capacity of stimulating the IL-4 production and the Th2 response. Interestingly, the Th2 immune response increased the susceptibility to the disease, whereas the Th1 immune response increased resistance to leishmaniasis [69]. CPB was also demonstrated to be involved in the inhibition of antigen presentation through the degradation of MHC class II molecules in the parasitophorous vacuole. Recently, other *L*. CPB mechanisms of action were elucidated, but their detailed description is beyond the scope of this review [70].

#### 2.2.6 Dihydrofolate Reductase-thymidylate Synthase (DHFR-TS) and Pteridine Reductase 1 (PTR1)

As already assessed for malaria and bacterial infections, in recent years, targeting folate enzymes has increasingly been recognized as a promising strategy for the treatment of parasitic infections [71-73]. Trypanosomatids are auxotrophic for folate and other pterinic compounds, essential factors in the biosynthesis of nucleic acids and proteins. To survive, these parasites have developed a biochemical supply and reuse of these factors from the infected hosts, exploiting two NADPH-dependent key enzymes, namely the dihydrofolate reductase (DHFR) and pteridine reductase 1 (PTR1) (Fig. **1**). Following the intake, pterins (such as folate or biopterin) are reduced twice, forming the active tetrahydro derivatives THF and THB, respectively. When DHFR occurs through conventional antifolates, such as MTX, the parasites overexpress PTR1, leading the enzyme to also process folate in sufficient THF levels, enabling them to survive. Keeping in mind the PTR1 metabolic bypass, the dual inhibition of both DHFR and PTR1 enzyme activities is strongly warranted for more efficient management of these parasitic diseases [72, 73].

The present review covers the last five years of literature on benzimidazole-based compounds endowed with the most promising antimalarial, antileishmanial, and antitrypanosomal profile worth of further improvements.

## 3 BENZIMIDAZOLE-BASED COMPOUNDS ACTIVE AGAINST MALARIA, LEISHMANIASIS, AND TRYPANOSOMIASIS

Resembling the main chemical features of the drug pentamidine, clinically used as therapy for African Trypanosomiasis and antimony-resistant leishmaniasis, Farahat *et al.* pursued their studies on the antiparasitic activity of bichalcophenes, developing a new series (Table **1**) in which a benzimidazole or indole moiety was introduced within a three aromatic spacer to outdistance the two terminal amidine groups [74].

Dicationic bisbenzamidines and structurally related heterocyclic analogues have long been known as parasitic DNA-targeted non-covalent agents [38].

In this regard, Farahat *et al.* [74] showed the benzimidazole diamidines strongly targeting the DNA minor groove by the analysis of circular dichroism (CD) spectra, and compound **1a** providing nanomolar potency against *P. falciparum* and a high selectivity index (SI) (Table **1**). Indeed, a better biological profile was exhibited by the indole isosteres **1b** and **1c**, for which a superior SI was noted (Table **1**). In the following step, the indole diamidines were tested *in vivo* using a mouse model which recapitulated the acute hemolymphatic phase of African trypanosomiasis; interestingly, the 2-[5-(furan-2-yl)thiophen-2-yl]indole derivative (**1c**) demonstrated to cure all T.b.r. infected mice at a dose minor than that of pentamidine.

Also, the diphenyl-based bis(2-aminoimidazoline) and bisguanidine DNA binders were previously identified as potent antiprotozoal agents against *Plasmodium spp* and trypanosomatids (*T. brucei*, *T. cruzi*, *Leishmania*) [39, 75]. On the wave of these results, monocationic 5-guanidinobenzimidazoles were investigated as broad-spectrum antiprotozoal agents [76], showing to be more efficacious and safer against *P. falciparum* with respect to *T. brucei* (see cpd **2a** and **2b**, Table **2**). No significant activity was observed against *T. cruzi* and *L. donovani* parasites. In particular, bulky aromatic rings, linked to position 2 of the 5-guanidinobenzi-midazole scaffold, were identified as a common signature for the best performance of this class of compounds, as for the rank-ordered list: biphenyl, 4-(3,4-dimethoxy-phenoxy)phenyl and bithiophene motifs. Docking studies disclosed the binding mode of compound **2a** to DNA minor groove of the d(CGCGAATTCGCG)_2_ duplex, where the guanidinium group formed a salt bridge and HB interactions with the phosphate moiety of G22 and the NH group of benzimidazole H-bonded to T19; the biphenyl ring concurred to stabilize the complex forming weaker aromatic HBs with T7, T8, T19 and T20. Molecular dynamics revealed that the DNA-ligand (**2a** and **2b**) interactions permitted a better stabilization of DNA conformation than its apo form, thus dampening its functional activity.

Fragment-based drug discovery (FBDD) strategies have achieved a wide consensus both in hit generation and hit-to-lead optimization process due to their potential to increase the chance of developing more targeted lead compounds that are small in size and, for this reason, highly ligand-efficient [77]. The application of this approach permitted the discovery of the benzimidazole derivatives as promising inhibitors of *P. falciparum* apical membrane antigen 1 (AMA-1) [45, 78], a protein responsible for the merozoite entry into the host erythrocytes. In an effort to identify novel *Pf* AMA-1 targeting benzimidazole agents, as a continuation of a previous study [78], R.S. Norton and P.J. Scammells identified a new series of 2-amino-1-(2-hydroxyphenyl)benzimidazoles (Table **3**). The novel compound library displayed a remarkable antimalarial activity against the 3D7 laboratory reference strain, especially when the phenol moiety bore electron-donating methyl and methoxy groups. The inclusion of the phenol group in a benzofuran ring abolished the activity, while its expansion to α-naphthol was tolerated without causing any significant improvement on the activity. The compounds showed to be non-toxic, thus providing a high therapeutic window, since the ratio of CC_50_ HEK293 to IC_50_ 3D7 was >10^3^.

The screening of antimalarial activity was also extended to the Cambodian Cam3.II^rev^ and the drug-resistant Cam3.II^C580Y^ clinical isolates and the compounds still reached nanomolar range potencies. In the evaluation of the cross-resistance profile, compounds **3a** and **3b** were shown to inhibit the replication of the Dd2 chloroquine-resistant strain, harboring distinct sets of point mutations in the Pf transporter (PfCRT), such as the crucial K76T and A220S [79], and the Dd2-SJ733 strain carrying resistance to the cation-transporting ATPase 4 inhibitor SJ733 [80]. This broad spectrum of activity *versus* notable examples of drug-resistant strains makes the case that they could act through an innovative mechanism of action, thus deserving further in-depth analyses. The preliminary *in vitro* and *in vivo* evaluation of the pharmacokinetic (PK) profile pointed out the need for a chemical optimization for this class of 2-aminobenzimidazoles to overcome the observed low metabolic stability and moderate-low intrinsic clearance.

Bhoi *et al*. [81] also enlightened the good antimalarial activity of a benzimidazole set (Table **4**), bearing in position 2 with a 2-phenol moiety decorated with electron-donating alkyl groups, as derived from the rearrangement of 2-formyl carvacrol. All the compounds were proven to be active against the Pf laboratory strain with IC_50s_ ranging from 1.52-3.31 µM. The 5,6-disubstituted derivatives **4c** and **4g** reached the potency of the standard drug quinidine (IC_50_ = 0.83 µM).

The *in-silico* investigation of the physicochemical properties of these compounds reported that no violation of Lipinski’s rule of five descriptors occurred and that they had a good PK profile (bioavailability score of 0.55). The analysis at molecular level of the most important features involved in the interaction of the tile compounds in complex with the *Pf* dihydroorotate dehydrogenase (DHODH) enzyme (PDB ID: 1TV5) showed their putative capacity of binding, albeit with some variations, to key residues of the active site, including Tyr528, Cys276, Phe278, and Ile263, as experienced by the reference compound chloroquine [81].

Singh *et al*. [82] developed a hybrid molecule (Table **5**) where a phthalimide-based hit compound was improved by the incorporation of a benzimidazole ring and a triazole moiety as pharmacophoric elements, recurrent in several examples of natural and synthetic compounds.

All the compounds were shown to impair the growth of the Pf3D7 strain with potencies in the submicromolar range and also of the CQ-resistant RKL-9 strain to a slightly lesser extent. Interestingly, no toxicity was observed against HepG2 cells (CC_50_> 100 µM), up to 500 µM against HEK2GST cells; hence, the corresponding SIs (ratio of CC_50_ to IC_50_(3D7)) were at least superior to 123 and 600 respectively (Table **5**). Moreover, no haemolytic activity was observed up to 5 µM on human red blood cells, advising a preferential selectivity for infected red blood cells with respect to normal blood cells. The effect of compounds on the specific blood stage of parasite growth was evaluated after 60 hours post-treatment, and defects in invasion and egress stages of the host red blood cells were observed [50]. The biochemical study of the mechanism of action revealed that these hybrid compounds promoted the depolarization of the mitochondrial membrane potential, causing mitochondrial dysfunction and subsequent cell death; additionally, compound **5b** showed to interact with the recombinant PfαI-tubulin (Kd= 26.4 µM) from *E. coli* strain BL21, altering microtubule dynamics. The *in vivo* antiparasitic activity was tested in BALB/c mice infected with *P. berghei*, and all derivatives demonstrated to significantly decrease the parasite load compared to an untreated control group on day 7 post-infection.

Pyrido [1,2-*a*]benzimidazole was advised as a promising core structure for the development of antimalarial agents [83], which were successively demonstrated to impair the heme detoxification process, leading to non-toxic hemozoin thus reducing parasite growth [48, 49, 84].

Sousa *et al*. [85] identified three potent pyrido [1,2-*a*]benzimidazole inhibitors **6a-c** endowed with comparable *in vitro* activities against 3D7 and CQ-resistant W2 strains (Table **6**). In particular, **6a** was found to be the most effective, and so it was selected for addressing further studies on the mechanism of action, using some conventional drugs for the purpose of comparison. The compounds were shown to kill both ring and trophozoite stages of the parasite, similarly to CQ and AQ, thus guaranteeing a long-lasting activity, and, unlike what happens for AQ, they were not susceptible to GSH conjugation. They exhibited significantly reduced parasitaemia in mice infected by *P. berghei*, surpassing the effectiveness of CQ. *In vitro* and *in vivo* studies corroborated **6a** binding to the cytosolic heme pool and heme-dependent toxification effects, that, however, occurred only after prolonged exposure to the compound; thus, the implication of other unexplored targets was suggested to justify the rapid parasiticidal activity. Interestingly, the compounds displayed a preferential ability to enter parasite cells more rapidly than host cells, thus exhibiting a species-selective tropism.

Nieto-Meneses *et al.* [86]. reported a series of N-benzyl-1H-benzimidazol-2-amines, among them compounds **7a** and **7b** (Table **7**), resulted in more activity against *L. mexicana* cutaneous amastigotes and less cytotoxicity against murine macrophage cell line J774.2 than miltefosine, exhibiting an higher SI with respect to reference drugs (Table **7**) (SI = 6.5 for amphotericin B).

The compounds were characterized by a methyl group at N-1 of the benzimidazole ring, an oxygen atom at the C-2 position of the benzyl portion (hydroxyl or methoxyl for **7a** and **7b**, respectively) and no substituents in the benzenoid moiety; the modification of these main structural features led to less active analogues. Based on the encouraging results, the activity of the two compounds was also evaluated against the promastigotes and amastigotes of various *Leishmania* species. In detail, only benzimidazole **7a** was slightly less effective than miltefosine against *L. braziliensis* mucocutaneous promastigotes, whereas both derivatives were less active than reference compound against *L. donovani* visceral promastigotes (Table **7**). Regarding intracellular amastigotes, they were more active than both miltefosine and amphotericin B (IC_50_ 0.87 µM) against *L. mexicana,* showing high SI values, whereas they exhibited lower activity than miltefosine against *L. braziliensis* and mostly *L. donovani* amastigotes.

To elucidate the benzimidazoles target, their ability to inhibit recombinant *L. mexicana* arginase (LmARG) was evaluated using the QuantiChom^TM^ Arginase Assay Kit in the presence of Nor-NOHA (N-Hydroxy-nor-L-arginine) as positive control. Interestingly, compound **7b** inhibited the activity of LmARG by 68.27%, resulting in about 3-fold more activity than **7a** (24.34% inhibition) and 2-fold more activity than Nor-NOHA (32% inhibition); interestingly, **7b** did not inhibit the activity of human arginase but stimulated it by 24.21%. An *in silico* docking study was performed, and compound **7b** was the best ranked with a score of -5.5 kcal/mol with respect to -4.46 kcal/mol of its analogue **7a**. Based on calculated physicochemical descriptors, both compounds fulfilled all Lipinski’s and Veber’s rules, prospectively offering a good bioavailability profile.

As result of a preliminary screening on their *in-house* database, De Luca *et al*. [87] reported four 1,2-disubstituted-1*H*-benzo[*d*]imidazole derivatives **8a-d** (Table **8**) as effective inhibitors (97.4-92.0% inhibition at 20µM) of *L. mexicana* CPB2.8∆CTE without inducing toxicity to similar human cysteine proteases cathepsin-B (no inhibition) and cathepsin-L (maximum of 20% inhibition).

The three compounds **8a-c,** characterized by an electron-rich sulfone bridging moiety between N1 of the benzimidazole nucleus and the phenyl ring, resulted in more activity than their analogue **8d** bearing a methylene group as linker at the same position. The presence of an electron-withdrawing group in the phenyl ring of the 2-thioacetamide portion, as well as the introduction of an additional substituent to the C-6 position of the benzimidazole core, positively influenced the activity (**8a** *vs.* **8b** and **8b** *vs.* **8c**). The cell-based assay using *L. infantum* intracellular amastigotes and miltefosine as a positive control (IC_50_ 4.5 µM) revealed an opposite behavior of the compounds against the whole organism, being **8d** more active than its analogues **8a-c**. Benzimidazole **8d** was also the most cytotoxic (CC_50_ 8.0 µM) in primary peritoneal mouse macrophages (PMM). The main structural determinant between compounds **8a-c** and **8d** is the sulfone group, which, as a strong H-bond acceptor, probably promotes the binding to the catalytic site of the target enzyme, the cysteine proteinase CPB2.8∆CTE, but negatively affects the passive diffusion by the biological barriers due to its polar nature. *In silico* studies by using the homology model of mature *L. mexicana* CPB2.8∆CTE were reported. The calculated K_i_ values obtained by noncovalent redocking for compounds **8a-d** followed the same trend as the experimental values, whereas the ligand efficiency (LE) values showed that derivatives **8a,d** (LE 0.28) were slightly more powerful than analogues **8b,c** (0.26 and 0.25, respectively). The best-docked pose of benzimidazoles **8a,d** pointed out their different accommodation on the enzymatic surface based on their substituents pattern. The binding with the enzyme exploited almost all H-bond interactions, although one π-H interaction for compound **8c** between the imidazole and the hydrogen of Asn162 and three π-π interactions for compound **8d** between the imidazole and the pyrrole of Trp185 and between the ring of the benzimidazole and the pyrrole or the phenyl of Trp185, were also reported. The compounds did not fulfill the whole Lipinski’s rule, whereas during *in silico* ADME studies, they exhibited a promising oral availability with a human intestinal absorption >96% but low Caco-2 permeability (48.11-11.92%) and an efficacious plasma protein binding (PPB = 100). To investigate the preclinical value of these compounds, the Authors performed Ames’s test. Except for derivative **8c**, they resulted in no mutagen, and **8a** and **8d** were also noncarcinogenic in rats and mice.

**Fig. (2):**
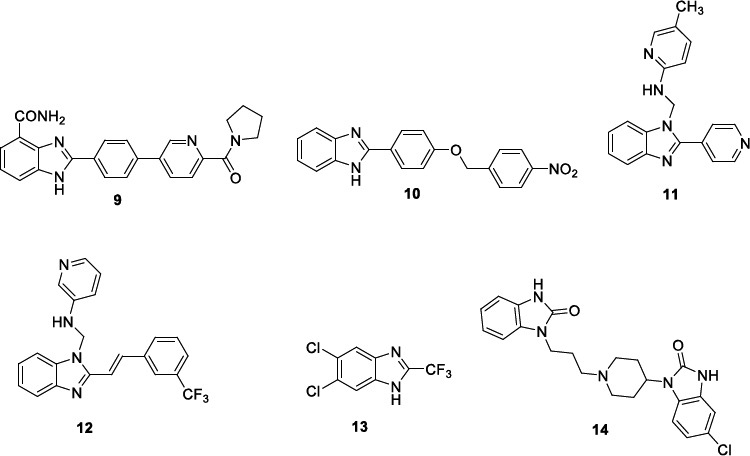
Structures of benzimidazoles **9-14** endowed with anti-leishmanial activity.

Kumar *et al*. performed a virtual screening of 221 compounds from several online databases (PubMed, ChEMBI, DrugBank) by using the predicted TryR structure [88]. Fifty-five compounds that resulted from the screening were able to bind the enzyme, and among them, 7 satisfied the docking score threshold of -7.00 kcal/mol. Among them, the benzimidazole derivative **9** (Fig. **2**) showed *in silico* good oral bioavailability and no toxicity. The compound strongly interacted with the TryR with a docking score value of -9.7 kcal/mol, and MD simulations enlightened a stable ligand-protein interaction. Enzymatic and cell-based assays on **9** revealed a TryR dose-dependent inhibition and an anti-leishmanial activity against *L. major* cutaneous promastigotes with an IC_50_ of 44.10 µM, although 3-fold less active than mitelfosine (IC_50_ 13.92 µM), without evincing a significant effect on RAW 264.7 macrophage cells viability. The cytotoxic effect of benzimidazole **9** on *L. major* promastigotes led to an increase of cells in apoptosis and necrosis with respect to the control and morphological changes in the parasites, such as cell size decrease, flagellar size reduction and cell membrane bleeding. Interestingly, compound **9** induced a dose-dependent NO release in RAW 264.7 macrophage cells, and *in vivo* experiments caused a significant reduction of parasite load and foot pad lesion size.

Kapil *et al*. [89] performed two receptor ligand pharmacophore models for homology-modelled dihydrofolate reductase-thymidylate synthase (DHFR-TS) and pteridine reductase 1 (PTR1) (PDB ID: 1E7W) with the aim of designing dual acting antileishmanial inhibitors. In detail, the generated DHFR-TS model showed four main structural features: one hydrogen bond acceptor (HBA), three hydrogen bond donors (HBD), one hydrophobic (HY) and one negative ionizable (NI) with a selectivity score of 14.289, whereas the generated PTR1 model exhibited six main structural features: one HBA, two HBD, one HY, two aromatic rings (RA) with a selectivity score of 11.429. The two models were used to screen an *in-house* database, and 5 benzimidazole derivatives were identified as hits. Compound **10** was mapped on the two validated models, and some overlap emerged: NH of benzimidazole on HBD feature, benzyl group on RA/HY features, the benzyl substituents on HBA and NI features. Benzimidazole **10** showed some similar structural characteristics, with derivative **9** bearing both a phenyl ring at the C-2 position of the benzimidazole core and a pyridyl/phenyl ring decorated in the *para* position with a polar substituent. Docking studies demonstrated the binding of compound **10** with the active site of both targets and MD simulation, the formation of a stable complex between **10** and the two proteins. The low MM-GBSA binding energy for human thymidylate synthase (1JU6) *versus* DHFR-TS and PTR1 pointed out the valuable selectivity of compound **10**. The anti-leishmanial activity of benzimidazole **10** was evaluated against *L. donovani* promastigotes (IC_50_ 68 µM), resulting in about 6-fold less activity than miltefosine (IC_50_ 12 µM). Compounds **11** and **12** (Fig. **2**), bearing at N-1 of the benzimidazole core a pyridinylmethyl moiety, were reported by Patel *et al*. [90]. Both compounds showed antiprotozoal activity against *L. mexicana* (**11**, IC_50_ 1.36 µM; **12**, IC_50_ 0.66 µM) and *T. cruzi* (**11**, IC_50_ 3.38 µM; **12**, IC_50_ 3.84 µM). Interestingly, compound **11** was 2-fold more active than miltefosine (IC_50_ 1.35 µM) against *L*. parasite, and both compounds were about 3-fold more active than benznidazole (IC_50_ 11.14 µM) against *T*. protozoan. Benzimidazole **11** fullfilled all the Lipinski’s, Veber’s, Ghose’s and Egan’s rules, whereas compound **12** showed one violation in Ghose and Egan's filter screening. Pharmacokinetic properties for both compounds were predicted, showing a high gastrointestinal absorption. The compounds inhibited several cytochromes; **11** also exhibited blood-brain barrier (BBB) permeability and was a P-glycoprotein (Pgp) substrate. Moreover, both derivatives were not cytotoxic and hepatotoxic but carcinogenetic and mutagenetic and **12** also immunotoxic.

Sánchez-Salgado *et al*. [91] reported an interesting chemoinformatic approach using the ChEMBL database along with an additional literature search to recover the benzimidazole nucleus as a target scaffold. About 235 benzimidazoles were identified with activity against several *L.* species. Most of the compounds were active against *L. major* and *L. donovani* followed by *L. infantum*; a moderate number of derivatives showed activity against *L. mexicana* and few compounds against *L. amazonensis* and *L. braziliensis*. In detail, 54% of compounds were active in visceral (*L. donovani* and *L. infantum*), 44.3% in cutaneous (*L. major, L. mexicana* and *L. amazonensis*) and 1.7% in mucocutaneous leishmaniasis (*L. braziliensis*). The activity of benzimidazoles deeply varied based upon the different substitution patterns, where the 1,2,5 and 6 positions on the benzimidazole core resulted relevant for the Structure-Activity Relationship (SAR) studies. Hence, the authors screened the benzimidazole **13** (Fig. **2**), previously tested against *L. mexicana* promastigotes (IC_50_ 20.80 µM), *versus* other *L.* species [92]. In particular, compound **13** resulted in more activity against *L. amazonensis* promastigotes (IC_50_ 4.11 µM) than miltefosine (IC_50_ 9.56 µM) and worked similarly to miltefosine against *L. infantum* promastigotes (IC_50_ 8.63 µM and 7.42 µM, respectively). Furthermore, the compound was screened against the intracellular amastigote stage of leishmania parasites, which reproduces a more physiological and relevant model of the disease in humans. The compound retained its activity but to a lesser extent than miltefosine.

Interestingly, only a few benzimidazole derivatives were reported as active against *L. amazonensis* in the literature so far. The investigation of the mechanism of parasite inhibition allowed to observe an alteration of the tubulin-microtubule equilibrium in *L. infantum* promastigotes when incubated with an anti-*β*-tubulin antibody and compound **13**. Moreover, in *in vitro* studies **13** showed a strong decrease in parasite load in the infected macrophages of both species, with particular regard to *L. amazonensis* infections.

Recently, immunotherapy has led to interesting results in the treatment of CanL by using compounds able to sustain the host immune response against the infection. Among these agents, the benzimidazolone domperidone **14** (Fig. **2**), a dopamine D2 receptor antagonist, was approved (Leisguard^®^) in the prevention and treatment of CanL alone or in combination with conventional anti-Leishmania chemotherapy. The compound is able to potentiate the phagocytic cell activity involved in the Th1 immune response due to a release of serotonin, which increases the prolactin blood concentrations. Prolactin is a proinflammatory lymphocyte-derived cytokine that stimulates T CD4^+^ lymphocytes, the release of cytokines, such as IL-2 and IL-12, IFN-γ and TNF-α, thus activating the NK cells and macrophages, and causes a decrease of CD4^+^ Th2 cytokines and TNF-β [93, 94]. As mentioned above, Th1 immune response enhances the resistance to leishmaniosis [69]. Very recently, Cavalera *et al.* reported the role of domperidone in ameliorating kidney functions in leishmaniotic dogs affected by chronic kidney disease (CKD) by maintaining stable serum creatinine and by reducing anti-*L. infantum* antibody titres, globulins, gamma globulins, and C-reactive protein [95]. The same authors reported the efficacy of domperidone combined with a renal diet to slow the progression of nephropathy in leishmaniotic dogs with CKD [96].

Ferreira *et al*. [97] carried out a hit-to-lead optimization study starting from the 2-aminobenzimidazole **15a** (Table **9**) emerged as a hit in a GlaxoSmithKline high-throughput screening (HTS) diversity set of 1.8 million compounds against *L. donovani*, *T. cruzi* and *T. brucei* by using whole-cell phenotypic assays. Compound **15a** showed moderate activity against *L. infantum* without affecting mammalian cells and good permeability [98]. However, it exhibited high lipophilicity and low metabolic stability in mouse liver microsomes (MLM) due to oxidative reactions and subsequent glucuronidation on the propyl chain at N-1 of the benzimidazole ring. An exhaustive optimization study on hit **15a** clarified the key structural features endowed with beneficial effects on the anti-leishmania profile. In detail, the presence of electron-withdrawing groups (EWGs) on the phenyl ring of the benzimidazole core increased the metabolic stability, whereas the addition of a nitrogen atom increased the cytotoxicity. Among the EWG substituents, the hydrophobic ones maintained the activity, whereas the polar ones jeopardized the activity. The bioisosteric replacement of the phenyl ring linked to the amide fragment with a pyrazole and a pyridine nucleus had a favourable effect on the potency and metabolic stability. The presence of a hydrophobic chain on the N-1 of the benzimidazole was essential for the activity and improved the metabolic stability in the case of branched and less lipophilic alkyl groups. The results of this SAR study led to the benzimidazole compounds **15b-e** exhibiting a better activity, an excellent metabolic stability and a lower lipophilicity than hit **15a** (Table **9**).

Selected compounds **15a** and **15d** were then tested against the amastigotes of several VL and CL strains and showed good activity with an IC_50_ range of 0.10-24.82 µM.

*In vitro* ADME data demonstrated that all analogues **15a-e** had a comparable mouse and human microsomal stability and good passive permeability as well as plasma stability and protein binding capacity. Derivatives **15d** and **15e** evinced poor aqueous kinetic solubility. To investigate the safety profile of this class of compounds, inhibition of *h*ERG and cytochrome P450 isoforms was assessed. All benzimidazoles **15a-e** weakly inhibited the *h*ERG channel and the cytochrome P450 family enzymes and did not interfere with the activity of key human enzymes and receptors, such as PDE3A, PDE4B2, 5-HT1A, B2 bradykinin, as an example. Compounds **15a** and **15d**, showing the best PK profiles *in vivo* with good bioavailability and plasma half-lives, were tested in an acute VL mouse model using an *L. infantum* strain. Unfortunately, both compounds lost their activity, probably due to the low systemic exposure and the poor safety profile.

Tonelli *et al*. [99] developed two series of benzimidazole derivatives, which proved to be active against *L. tropica* and *L. infantum* (Table **10**). The first series bore a long alkyl chain and an ammonium moiety in position 2 of the benzimidazole to mimic the structure of miltefosine, while the second series was characterized by 2-phenyl/2-benzyl benzimidazole scaffold substituted in position 1 with a basic chain resembling that of sitamaquine. More than 75% of the compounds in both series were dual inhibitors of *L. tropica* and *L. infantum* promastigotes. Generally, the quaternization of 2-alkylbenzimidazoles improved the activity, which fell in the low micromolar/submicromolar range; in particular, compound **16a** displayed 228- and 93-fold lower IC50s than those of miltefosine against both Leishmania species (Table **10**). However, the cytotoxicity linearly increased with the potency, so the SI was low *versus* HMEC cells but superior *versus* Vero76 cells. Among the compounds featured by a basic side chain, the 1-quinolizidinylmethyl derivatives were more potent than dialkylaminoalkyl ones, and compound **16b** resulted in being the most potent (Table **10**). Also, this compound manifested some degree of toxicity against HMEC cells, thus showing low SI values, but was not toxic against Vero76 cells (CC_50_> 100 µM). The hit compounds **16a** and **16b** were then evaluated against the intramacrophagic amastigote stage of *L. infantum*, demonstrating for compound **16a** an IC_50_= 0.31 µM, about 3-fold superior to that of miltefosine, while for compound **16b** an IC_50_= 4.76 µM. Therefore, further exploration of the chemical space around the 2-alkyl and 2-benzyl benzimidazole scaffold may be worthy of consideration.

Recent studies have detected the naphtoquinone scaffold as a starting point for the development of novel antiprotozoan compounds. In particular 2-aryloxynaphtoquinone-based compounds have shown an interesting inhibitory profile towards *T.cruzi* [100]. Adding to the growing interest towards the benzimidazole scaffold as the main pharmacophoric unit of several molecules endowed with antiprotozoan activity, the bioisosteric substitution of the benzene fused ring of the naphtoquinone core with an imidazole ring has been explored [101].

The antiprotozoan activity of these compounds was evaluated *in vitro* towards the epimastigote form of *T. cruzi*, Dm28c strain (Table **11**). The non-infective epimastigote form could be found in the midgut of triatomine and acquire the ability to infect mammalian hosts only after its conversion to metacyclic trypomastigote. All the benzimidazole-quinone compounds manifested higher antiparasitic activity than the reference compound nifurtimox. Compounds **17a-d**, characterized by the phenoxy- moiety in R^1^, appeared to be the most active with IC_50_ values in the 1.42-4.07 μM range. Compounds **17a-d** were subsequently tested against the trypomastigote infective form of *T. cruzi* (Dm28c strain), showing even better inhibitory potency, ranging from 0.65 to 1.39 μM and a promising selectivity index > 10.

In order to assess the mechanism of action of this class of compounds, the target identification was attempted by means of molecular modelling studies through induced-fit docking (IFD) methodology. *T. cruzi* GAPDH, TryR, and OYE were selected as putative targets due to their involvement in the mechanism of action of some naphthoquinone-based multitarget antiparasitic compounds [100, 102, 103]. Indeed, on the basis of the calculated docking scores, only TryR and OYE resulted to be probable target enzymes of this series.

The IFD of the previously studied naphthoquinones on TryR (PDB code: 1BZL) allowed the identification of the Z-site of this enzyme as the potential binding site due to the interaction of the compounds with a hydrophobic region formed by Phe396, Pro 462, and Leu399 residues. The best compound, **17a,** showed high affinity to TryR with a docking score of - 7.905 kcal/mol, involving H-bond interactions between the quinone carbonyl groups and Lys62 and Ser464; the N3 of the imidazole ring with Lys62 and the oxygen atom of the phenoxy group with Asn433. Additionally, hydrophobic interactions between the phenoxy group and the residues Leu63, Leu399 and Phe396 concurred with the stabilization of the complex. The comparison between the complex of the imidazoquinone **17a** and its naphthoquinone analogue pointed out the key contribution of N-3 of the imidazole ring for the inhibition of TryR and the observed *in vitro* activity towards *T. cruzi.*

Benzimidazole-quinone **17a** also showed a strong binding affinity with OYE (docking score of - 7.905 kcal/mol), smoothly fitting a binding site close to the FMN prosthetic group (PDBCode: 3ATZ): both H-bond interactions by its CO groups of the quinone moiety with Tyr364, Asn198, His195 of OYE and hydrophobic, and π-π stacking interactions of the phenoxy ring with Phe71 and Tyr200 were observed. However, further studies are needed to provide experimental evidence of TryR and/or OYE as the likely molecular targets of this class of benzimidazole-quinone derivatives [100].

Several inhibitors belonging to benzothiazole [104, 105], benzoxazole [106], and benzimidazole [107] chemical classes exhibited the ability to target the TIM enzyme. In this field, Vázquez-Jiménez *et al.* [108] detected novel benzimidazole compounds capable of interacting with the TcTIM interface region by performing a ligand-based virtual screening (LBVS) from the ZINC15 database. From a total of 67,141 benzimidazole-containing compounds, 1604 compounds were selected because of their binding energy values between -10.6 and -8.9 Kcal/mol and adherence to Lipinski rules. These compounds were subsequently clustered into ten groups considering the types of interactions in the complex with the enzyme. Only two compounds, **18a** and **18b**, were thus tested against blood trypomastigotes of the NINOA and INC-5 strains of *T. cruzi* to assess their trypanocidal activity (Table **12**).

*In vitro* determination of the half-maximal lytic concentration (LC_50_) confirmed that the trypanocidal ability of the compounds was comparable to that of the drugs nifurtimox and benznidazole. Molecular dynamic simulations of compounds **18a** and **18b** with *T. cruzi* triose phosphate isomerase showed that the **18b**-TcTIM complex is more stable than that of the control ligand L1 (structure not shown), which was previously described as a TcTIM inhibitor [109].

Regarding the SwissADME calculated physicochemical properties, the two compounds shared drug-like characteristics, with moderate water solubility, good intestinal adsorption, and no brain-blood barrier permeability. However, they seemed to be vulnerable to rapid hepatic inactivation and also to inhibit almost all the CYP450 isoforms (except for 1A2 isoform), thus suggesting a high risk of causing drug-drug interactions. Both **18a** and **18b** were predicted to be P-glycoproteins (P-gp) substrates, an efflux pump that is related to the risk of developing drug resistance.

Finally, docking calculations unravelled a preferential selectivity of **18b** for the trypanosomal isoform of the glycolytic enzyme, TcTIM (DGbind= -10.2 Kcal/mol), over the human isoform (HsTIM: DGbind= -5.9 Kcal/mol) [108].

Beltran-Hortelano *et al*. [110] screened against *T. cruzi* some Mannich base-type derivatives, whose activity was previously related to the inhibition of the trypanosomal superoxide dismutase, a Fe-containing metalloprotein (Fe-SOD) [111]. The library was categorised into different groups (series a-g) according to common structural features. The only benzimidazole-containing cluster was series b, composed of 17 compounds, among which **19a** and **19b** demonstrated good antiparasitic activity against *T. cruzi* CL-Lu: Neon clone epimastigotes (Table **13**). However, no further studies were performed about them due to the high toxicity demonstrated against BSR mammalian cells.

As mentioned above, for the benzimidazole-based antimalarials, the pentamidine amidine motif was also explored in the search for novel DNA-binding anti-trypanosoma agents and even better broad-spectrum antiparasitic compounds. In this context, Bistrović *et al*. [112] synthesised a series of 5-amidino benzimidazoles as anti-bacterial and anti-protozoal compounds, characterized by a hydrophobic 1-aryl-1,2,3-triazole moiety.

These compounds were tested *in vitro* for the antiparasitic activity against *T. brucei* bloodstream form (strain 221) and toxicity against the mammal L6 cell line (Table **14**). They inhibited *T. brucei* in the low micromolar range (IC_50_= 1.1 to 1.6 μM). These compounds characteristically share a *p-*methoxy phenyl moiety on the triazole ring and differ from each other only for the structure of the amidino motif. Notably, the best inhibitor, **20b**, proved to be 4-fold more potent than the reference compound Nifurtimox. The insertion of other groups on the phenyl ring or other types of aromatic moieties negatively affected the antiparasitic activity, which reduced or even abolished. The binding affinity of the compounds to the nucleic acids, observed by UV/vis spectroscopy, was linearly correlated with the anti-microbial activity but not with the antiprotozoal one, thus reasonably assuming that other pathways and targets could also be involved in the mechanism of action.

The class of 2-aminobenzimidazoles was identified by high-throughput screening (HTS) technology applied to a compound library of GlaxoSmithKline (GSK) in order to develop novel libraries of molecules targeting leishmaniasis [97] and trypanosomiasis [113, 114]. Subsequent hit optimization studies explored antiparasitic potency, cytotoxicity, adsorption, metabolism, and excretion as parameters for the progression of the most promising derivatives. The initial profiling of the hit compound **21a** confirmed its potency towards *T. cruzi* and *T.brucei*, no observable toxicity on HepG2 and MRC-5 mammalian cells (*T. cruzi strains* IC_50_ = 0.63 and 1.26 μM; *T. b. brucei* IC_50_ = 2.0 μM; MRC-5 CC_50_ > 64 μM; HepG2 CC_50_ >100) and good permeability, but a rapid *in vitro* clearance in mouse liver microsomes (MLM) and a high LogD (eLogD =4.6). The hit-to-lead and lead optimization programs focused on the substitution of the benzimidazole ring in positions 5 and 6, on the variation of the N-alkyl chain and of the acyl fragment linked to the 2-amino group.

McNamara *et al.* observed that the *p*-Cl substitution of the phenyl ring was an important requisite for the activity, as its substitution with stronger electron-withdrawing CF_3_ or OCF_3_ groups only produced a negligible improvement. The replacement of the phenyl ring with biphenyl, naphthyl rings or a long aliphatic chain still provided equipotent compounds to **21a**. A comparable SAR trend was observed against *T. brucei,* whereby the effect of the substituents in the diverse positions of the phenyl ring was similar for almost all the entries (mean IC_50_ 3.5 μM).

The chemical space around the N1-alkyl chain of the benzimidazole ring was extensively explored, starting from the N-propyl chain of **21a**. Analysing the *in vitro* IC_50_ values towards *T. cruzi*, the propyl chain proved to be the best choice since its shortening or removal was responsible for the loss of activity, whilst its elongation, as in the butyl and pentyl chains, led to a slight improvement of the potency but to a higher lipophilicity. Regarding *T. brucei*, the chain length variation did not affect the activity (IC_50_ 3.1-4.4 μM). The variation of the nature (ureido and reverse amide groups) and the length of the amide linker on C2 of the benzimidazole ring was inspected in another series without observing any substantial improvement over the phenylacetamide moiety of the prototype **21a**.

Finally, only the inclusion of halogen atoms (F, Cl and Br) in the 5 or 6 position of the benzimidazole ring allowed for more potent derivatives than **21a** (Tables **15** and **16**). However, the cytotoxicity parallelly increased, leading to a low therapeutic window. Analogous trend was observed in the case of *T.b. brucei.*

A follow-up study was published by Rezende Júnior *et al.* [114], where novel benzimidazoles structurally related to hit **21a** were synthesized and tested for their activity against *T. cruzi*, and for their ADME-Tox properties. Keeping the 1-alkyl-2-acylamino benzimidazole as the scaffold, diverse decorations in the 5/6 positions of the benzimidazole ring, 1-alkyl chains, and 2-acylamino fragments were introduced. As priority criteria for the progression of the molecules, a high potency and an adequate ADME-Tox profile were selected (IC_50_ < 5 μM, selectivity index (SI) > 30, MLM clearance < 30 μL/min/mg and LogD close to 3).

In order to modulate the overall lipophilicity of the compounds, the phenylacetic fragment underwent several modifications, introducing picolinic and nicotinic acid moieties, which allowed to retain good IC_50s_ generally lower than 10 μM. Regarding the 1-alkyl moiety, the trifluoroethyl chain was identified as the best substitution to balance the potency and the metabolic stability (**21g**) (Table **17**).

Unchanging the 6-CN and the 1-trifluoroethyl chain, some alternative heteroaromatic acylamines were sampled. The trifluoromethylpyridine derivative **21h** and compounds **21i** and **21j** bearing the trifluoromethyl pyrazole rings showed improved potencies and good metabolic stability, low lipophilicity, and high selectivity indexes (Table **17**).

The selected compounds **21g-j** were then evaluated against *T. cruzi* amastigotes, which exhibited a concentration- and time-dependent antiparasitic activity. Interestingly, differing from the traditional antiparasitic agents which are considered fast killers, these compounds could behave as slow killers since they require more cycles of parasite replication to exert their antiparasitic activity. The study of physicochemical properties pointed out that these compounds possess good permeability but low kinetic solubility values at pH 7.4 and 2.0. Whilst cytotoxicity against the host cell line MRC-5 used in the first screening was generally low (mean CC_50_ >50 μM), it increased against HepG2 and PMM cells. From the analysis of the risk for off-target adverse events, no significant evidence of activity was observed against a panel of key human enzymes. Thus, the compounds warrant a deep investigation of their promising safety profile.

## CONCLUSION

The management of protozoan diseases still represents a challenging task in WHO programmes, strengthening the need for solving the gap in innovation and more adequate investments in basic research and product development and new vector control strategies and technologies. The rapid global expansion of travel and trade, the growing interactions between humans and the animal reservoirs of pathogens and vector species, and the emerging environmental problems make the scenario very complex. Additionally, the report of natural coinfections involving diverse *Plasmodium* and *Leishmania* species poses a greater risk of morbidity and severe diseases, as the concomitant incidence of the two diseases in the same individual showed to jeopardise the host immune response [115]. Nevertheless, the impact of the COVID-19 pandemic has drastically shifted the priorities of public health. Thus, the prevention, diagnosis, and treatment strategies against protozoan diseases were unavoidably disregarded [116]. The existing drugs for human and animal vector-borne diseases are scarce and are affected by suboptimal efficacy, toxicity, and drug resistance. The development of novel antiprotozoal drugs may also take inspiration from the benzimidazole nucleus as a privileged pharmacophore in medicinal chemistry, thanks to its bioisosterism with naturally occurring purine nucleobases. The benzimidazole-based derivatives have demonstrated the ability to tackle distinct or even more multiple targets implicated in the pathogenesis of protozoan infections and have provided the potential for a broad spectrum antiparasitic activity. In this regard, the multitarget approach may be a valid tool to treat complex diseases like protozoan ones where a parasite, a vector (ectoparasite) and a host are strictly interconnected. New directions in the compounds’ design should focus on the identification of novel targets, especially those involved in the liver and transmission stages of the disease for a more meaningful blockage of the infection spreading to other human hosts. The SAR insights gathered through this survey offer the inspiration for developing more promising benzimidazole-based derivatives to treat the infections caused by Malaria, Leishmania, and Trypanosoma parasites. However, most of the proposed studies have stopped at the initial phases of drug discovery pipeline, limiting the biological evaluation at the enzymatic and cellular level, sometimes without identifying the molecular target. As only a few studies have explored the drug-likeness of these molecules, further investigations are warranted to translate them into therapeutics to handle these infectious diseases in humans and animals more effectively.

## Figures and Tables

**Table 1:** Structures and biological activity of bichalcophenes 1a-c against *P. falciparum* (P.f.) and *T. brucei* (T.b.r).

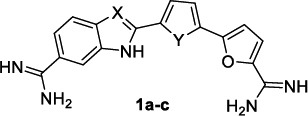				
**Compound**	**P.f. IC_50_^a^**	**SI^b^**	**T.b.r. IC_50_^a^**	**SI^b^**
**1a:** X = N, Y = O	91.9	454	102	409
**1b:** X = CH, Y = O	2.9	1000	15	1100
**1c:** X = CH, Y = S	6.8	4400	6.8	2588

**Note: **^a^IC_50_ (nM): compound concentration that reduced the parasite load by 50%; ^b^SI: selectivity index (IC_50_ L6/IC_50_ parasite).

**Table 2:** Structures and antiprotozoal activity of compounds 2a-c against *P. falciparum* (P.f.), *T. brucei* (T.b.r), *T. cruzi* and *L. donovani*.

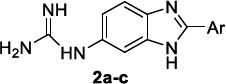								
**Compound**	**P.f. ** **IC_50_^a^**	**SI**	**T.b.r. IC_50_^a^**	**SI^b^**	**T.c. ** **IC_50_^a^**	**SI^b^**	**L.d. IC_50_^a^**	**SI^b^**
**2a,** Ar: 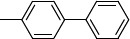	0.043	2360	51	2	130	0.8	126	0.8
**2b,** Ar: 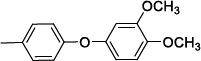	0.089	>1920	21	>8	100	>2	100	>2
**2c,** Ar: 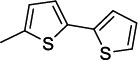	0.42	262	26	4.2	103	1.1	84	1.3

**Note: **^a^IC_50_ (µM): compound concentration that reduced the parasite load of 50%; ^b^SI: selectivity index (IC_50_ L6/IC_50_ parasite).

**Table 3:** Structures and antimalarial activity of benzimidazole derivatives 3a-e.

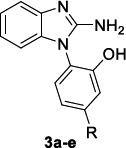					
**Compound**	**3D7 ** **IC_50_^a^**	**Dd2 ** **IC_50_^a^**	**^b^Cam3.II^rev^ IC_50_^a^**	**^c^Cam3.II^C580Y^** **IC_50_^a^**	**Dd2-SJ ** **Resistant ** **IC_50_^a^**
3a, R = H	77	97.3	350	264	46
3b, R = CH_3_	42	31.7	140	81	17.5
3c, R = OCH_3_	43	-	212	168	-
3d, R = 3,5-di-CH_3_	23	-	133	139	-
3e, R = 4,5-di-OCH_3_	6.4	-	47	63	-

**Note: **^a^IC_50_ (nM): compound concentration that reduced the parasite load of 50%. ^b^Cam3.II line has a *K*13 R539T mutation that is in Cam3.II^rev^ was gene-edited to wild type *K*13 gene. ^c^Cam3.II lines bearing the C580Y mutation in the *K*13 gene, which is responsible for resistance to artemisinin.

**Table 4:** Benzimidazole derivatives 4a-g bearing a 2-phenol moiety and their antimalarial activity.

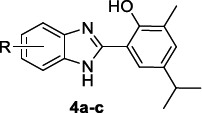	
**Compound**	**3D7 IC_50_^a^**
**4a**, R = H	3.31
**4b**, R = 6-CH_3_	6.28
**4c**, R = 5-CH_3_ 6-Cl	1.52
**4d,** R = 6-Cl	3.42
**4e,** R = 6-F	2.71
**4f**, R = 6-Br	3.19
**4g**, 5,6-diCl	1.85

**Note: **^a^IC_50_ (µM): compound concentration that reduced the parasite load by 50%.

**Table 5:** Example of benzimidazole phthalimide hybrids 5a-e and their antimalarial activity.

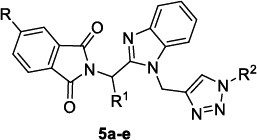						
**Compound**				**3D7 ** **IC_50_^a^**	**RKL-9 ** **IC_50_^a^**	**SI^b^**
**-**	**R**	**R^1^**	**R^2^**	-	-	-
**5a**	CH_3_	CH_2_CH(CH_3_)_2_	4-Cl-phenyl	0.74	1.71	>135 >675
**5b**	H	CH(CH_3_)CH_2_CH_3_	4-CF_3_-phenyl	0.64	1.62	>156 >781
**5c**	H	CH(CH_3_)CH_2_CH_3_	benzyl	0.81	1.88	>123 >617
**5d**	CH_3_	CH(CH_3_)CH_2_CH_3_	4-F-phenyl	2.2	3.43	>46 >227
**5e**	H	CH(CH_3_)_2_	4-CF_3_-phenyl	2.3	3.92	>43

**Note: **^a^IC_50_ (µM): compound concentration that reduced the parasite load by 50%. ^b^SI: selectivity index (CC_50_/IC_50_). *P. falciparum* 3D7 sensitive and RKL-9 CQ resistant strains.

**Table 6:** Antimalarial activity against the asexual blood stage of *P. falciparum* for compounds 6a-c.

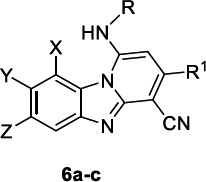							
**Compound**	**R**	**R^1^**	**X**	**Y**	**Z**	**3D7** **IC_50_^a^**	**W2** **IC_50_^a^**
6a	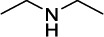	CF_3_	Cl	H	Cl	60.8	58.8
6b	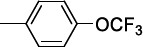	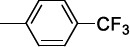	H	H	H	100.1	88.3
6c	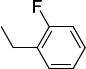	CF_3_	H	Cl	Cl	160.5	177.8

**Note: **^a^IC_50_ (nM): compound concentration that reduced the parasite load by 50%.

**Table 7:** Inhibition of several *L.* species proliferation for benzimidazoles 7a,b and miltefosine.

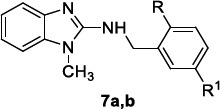						
**Compound** **7a, R = OH, R_1_ = Br** **7b, R = OCH_3_, R_1_ = H**	**7a**	**7b**	**Miltefosine**	**7a**	**7b**	**Miltefosine**
**Promastigotes IC_50_^a^**				**SI^b^**		
*L. mexicana*	2.62	3.21	15.34	91.76	317.75	10.23
*L. braziliensis*	54.05	127.7	48.37	4.88	9.61	3.25
*L. donovani*	198.47	223.8	114.68	1.33	5.48	1.37
**Amastigotes IC_50_^a^**				**SI^b^**		
*L. mexicana*	0.28	0.26	0.51	942.8	3939.2	307.8
*L. braziliensis*	34.22	33.78	22.67	7.71	30.32	6.92
*L. donovani*	222.23	218.24	66.74	1.18	4.69	2.35

**Note: **^a^IC_50_ (µM): compound concentration that reduced the parasite load of 50%; ^b^SI: selectivity index (CC_50_/IC_50_); CC_50_ from J774.2 cells.

**Table 8:** Structures, enzymatic and cellular activity of benzimidazoles 8a-d.

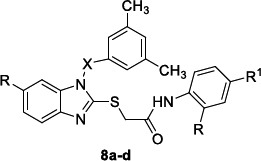						
**Compound**	**R**	**R^1^**	**X**	**CPB2.8∆CTE** **Inhibition (%)**	**(K_i_ µM)^a^**	***L. infantum* amastigotes IC_50_^b^**
**8a**	H	H	SO_2_	95.6	0.23	50.8
**8b**	H	CO_2_CH_3_	SO_2_	97.2	0.18	20.3
**8c**	Cl	CO_2_CH_3_	SO_2_	97.4	0.15	32.5
**8d**	Cl	CO_2_CH_3_	CH_2_	92.0	0.69	6.8

**Note: **^a^Assuming compounds as competitive inhibitors; ^b^IC_50_ (µM): compound concentration that reduced the parasite load by 50%.

**Table 9:** Anti-leishmanial activity, metabolic stability and lipophilicity of benzimidazoles 15a-e.

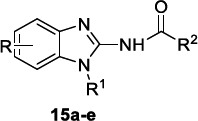						
**Compound**	**R**	**R^1^**	**R^2^**	***L. infantum* IC_50_^a^**	**MLM CI_int_ µg/min/mg**	**eLogD**
**15a**	H	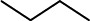	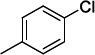	12	3094	4.6
**15b**	6-CN	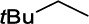	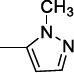	4.1	17	3.4
**15c**	6-CN	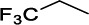	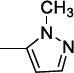	2.2	20	2.7
**15d**	6-CN	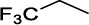	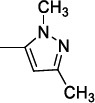	0.53	28	3.0
**15e**	6-CN	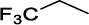	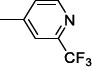	0.84	27	3.1

**Note: **^a^IC_50_ (µM): compound concentration that reduced the parasite load by 50%.

**Table 10:** *In vitro* data on antileishmanial activity against *L. tropica* and *L. infantum* and toxicity against the human endothelial cell line (HMEC-1) and/or monkey kidney cell (Vero76) of benzimidazole derivatives 16a,b and miltefosine.

**Compound**	**-**	**IC_50_^a^**	**SI^b^**	
	**-**		**HMEC**	**Vero76**
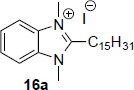	*L. tropica* promastigotes	0.19	4.10	30.5
	*L. infantum* promastigotes	0.34	2.29	17.1
	*L. infantum* amastigotes	0.31	-	-
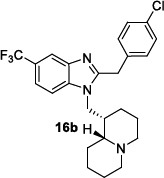	*L. tropica* promastigotes	3.70	4.58	>27
	*L. infantum* promastigotes	4.76	3.61	>21
**Miltefosine**	*L. tropica* promastigotes	43.26	2.3	-
	*L. infantum* promastigotes	31.26	3.2	-
	*L. infantum* amastigotes	1.05	-	-

**Note: **^a^IC_50_ (µM): compound concentration that reduced the parasite load of 50%. HMEC, human mammary epithelial cells and primate Vero76 cells.

**Table 11:** *In vitro* activity, cytotoxicity and SI on epimastigote and trypomastigote forms of *T. cruzi* of benzimidazole derivatives 17a-d.

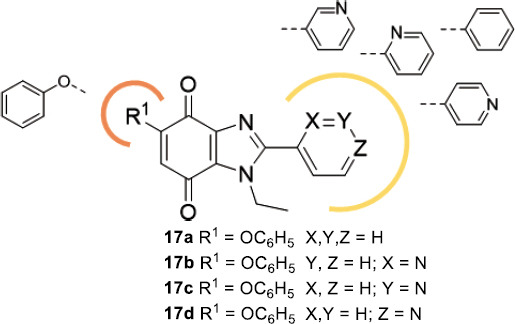				
**Compound**	**IC_50_^a^ Epim.**	**IC_50_^a^ Tryp.**	**IC_50_^a^ Vero cell**	**SI^b^ (Tryp.)**
**17a**	1.42 ± 0.07	0.65 ± 0.10	6.89 ± 0.50	10.6
**17b**	4.04 ± 0.30	0.88 ± 0.14	10.09 ± 0.70	11.5
**17c**	4.07 ± 0.27	0.96 ± 0.13	10.20 ± 0.79	10.6
**17d**	3.73 ± 0.32	1.39 ± 0.40	7.65 ± 0.66	5.5
**Nifurtimox**	21.05 ± 0.90	10.00 ± 0.40	>200	>20

**Note: **^a^IC_50_ (µM): compound concentration that reduced the parasite load of 50%; ^b^SI: selectivity index (IC_50_ Vero/IC_50_ parasite).

**Table 12:** Trypanocidal activity of compounds 18a,b.

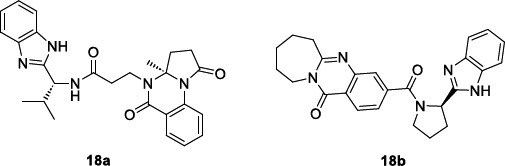			
**Compound**	**LC_50_^a^ NINOA**	**LC_50_^a^ INC-5**	**Docking Score (Kcal/mol)**
**18a**	155.86 ± 3.4	226.30 ± 15.4	-10.4
**18b**	179.55 ± 19.7	179.71 ± 19.0	-10.2
**Nifurtimox**	70.41 ± 8.0	139.37 ± 3.0	nd
**Benznidazole**	130.72 ± 8.8	191.28 ± 1.8	nd

**Note: **^a^ LC_50_ (µM) = half-maximal lytic concentration; nd = not determined.

**Table 13:** Antitrypanosomal activity against *T. cruzi* epimastigotes and cytotoxicity of derivatives 19a,b.

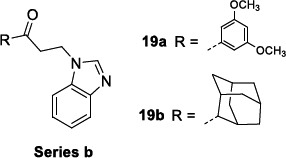			
**Compound**	**IC_50_^a^** **CL-Luc: Neon Clone Epim.**	**IC_50_^a^** **BSR Cells**	**SI^b^**
**19a**	11.1 ± 3.2	70.1 ± 9.6	6
**19b**	24.8 ± 5.8	67.6 ± 6.1	3
**Benznidazole**	3.8 ± 0.7	191.28 ± 1.8	67

**Note: **^a^IC_50_ (µM): compound concentration that reduced the parasite load of 50%; ^b^SI: selectivity index (IC_50_ BSR/IC_50_ parasite).

**Table 14:** Antitrypanosomal activity of compounds 20a-c against *T. brucei*.

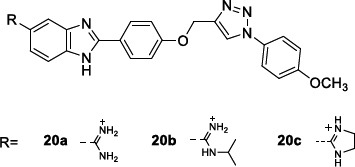		
**Compound**	**IC_50_^a^ *T. brucei***	**SI^b^**
**20a**	1.5 ± 0.3	200
**20b**	1.1 ± 0.3	>270
**20c**	1.6 ± 0.4	165
**Nifurtimox**	4.4 ± 0.7^b^	nd

**Note: **^a^IC_50_: compound concentration that reduced the parasite load of 50%; ^b^SI: selectivity index (IC_50_ L6/IC_50_ parasite); nd = not determined.

**Table 15:** Antitrypanosomal activity and SI of hit 21a against two different strains of *T. cruzi* and *T. brucei*.

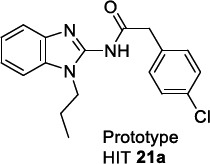		
***Parasite***	**IC_50_^a^**	**SI^e^**
***T. cruzi^b^***	0.63	79
***T. cruzi^c^***	1.26	40
***T. brucei^d^***	2.00	50

**Note: **^a^IC_50_: compound concentration that reduced the parasite load by 50%. ^b^*T. cruzi* primary growth inhibition assay performed with NIH-3T3 primary mouse embryonic fibroblast cells host cells. ^c^*T. cruzi* intracellular imaging assay performed with H9c2 rat embryonic cardiomyocytes. ^d^*T. brucei* primary growth inhibition assay in complete HMI9 medium. ^e^SI: selectivity index (CC_50_ mammalian cell line/IC_50_ parasite).

**Table 16:** Antitrypanosomal activity and SI of compounds 21b-f against *T. cruzi* and *T. brucei*.

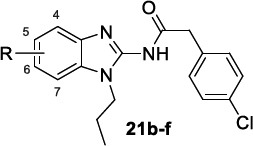					
**Compound**	**R**	***T. cruzi*** **IC_50_^a^**	**SI^b^**	***T.b. brucei*** **IC_50_**	**SI^c^**
**21b**	5-Cl	0.5	15.9	4.4	84%
**21c**	5-Br	0.79	>63.3	16	>5.18
**21d**	6-Cl	0.5	12.6	2.4	>34.6
**21e**	6-Br	0.4	32.5	1.5	>55.3
**21f**	6-F	1.3	19.2	0.90	15.5
**Benznidazole**		1.58	20	nd	nd
**Pentamidine**		nd	nd	0.0014	nd

**Note: **^a^IC_50_: compound concentration that reduced the parasite load of 50%; ^b^SI: selectivity index (CC_50_ H9c2 /IC_50_ *T. cruzi*); ^c^SI: selectivity index (CC_50_ HEK-293 /IC_50_ *T. brucei*); nd = not determined.

**Table 17:** Antitrypanosomal activity, metabolic stability and lipophilicity of benzimidazoles 21g-j.

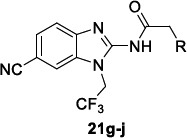					
**Compound**	**R**	***T. cruzi* IC_50_^a^ Tulahuen CL2 Strain**	**SI^b^**	**MLM CI_int_ µg/min/mg**	**eLogD**
**21g**	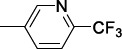	2.6 ± 0.66	>25	18	3.1
**21h**	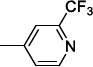	0.28 ± 0.30	>231	27	3.1
**21i**	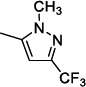	0.23 ± 0.96	34	28	3.0
**21j**	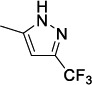	1.5 ± 3.19	>42	25	2.9
**Benznidazole**		2.1	nd	nd	nd

**Note: **^a^IC_50_: compound concentration that reduced the parasite load of 50%; ^b^SI: selectivity index (CC_50_ MRC-5 /IC_50_ parasite); nd = not determined.

## LIST OF ABBREVIATIONS

CL: Cutaneous
MCL: Mucocutaneous
VL: Visceral Kala-azar
DNDi: Drugs for Neglected Diseases Initiative
TPP: Target Product Profile
EMA: European Medicines Agency
LSF: Trypomastigote
SSF: Short Stumpy Trypomastigote Form
